# Health Tract, No. 48

**Published:** 1865

**Authors:** 


### To Parents, Guardians, and Youth.

Hundreds of thousands of dollars have been made out of boys from fourteen to twenty, and bachelors, by the authors of books on physiology, with plates to stimulate prurient curiosity to an ungovernable pitch. Hundreds of thousands of these publications are sold and given away. Scarcely any youth can read one of them without imbibing the impression that he is the victim of certain things, which unless promptly corrected will soon and surely lead to results of the most appalling character. We are in the frequent receipt of letters from mere boys, who have spent from five to five hundred dollars, without having derived a "particle of benefit from the treatment," and in terms of the most abject self-abasement and almost utter hopelessness, inquiring if it is too late for them to be saved. Letters come in from all parts of the country, inquiring if we know any thing of this, that, and the other one who has written such a book, or of some "company," "association," or "society," with benevolent names, whose advertisements are found in village newspapers all over the land. Some of our exchanges have them, which would not insert them at any price, if their true nature were known. It may save us the trouble of answering divers letters, and the cost of divers postage-stamps, envelopes, and sheets of paper, to state, that this subject is connected with the hours of sleep, and sixty pages are devoted to its consideration, and its only safe remedy, costing nothing but the exercise of a vigorous will, in the observance of certain specified habits and modes of life, in our book entitled "Sleep," or sent post-paid for $1.60. In very many cases the fears and imaginations of youth are so wrought upon that they are led to steal the money requisite to fee the harpies who wrote the books. We know an individual who in a very few years has laid up a hundred and twenty-one thousand dollars in this connection. The main portion of our book, however, is devoted to sleep proper; the importance of sleeping soundly, in a pure atmosphere, and how to do it; the advantage to old and young of sleeping alone, on single beds in large rooms; the injury to a family's thrift and happiness resulting from the mother having her rest broken by infant children, and how to remedy it, healthfully and happily, for all parties. We wish we were able to give one of these books on "Sleep" to every family in the nation, and place it on the shelf of all the libraries in Christendom, for certain are we that humanity would be happier thereby, and years would be added to the average of life, if the suggestions were carried out as to

1st. Securing sound, connected, and refreshing sleep every night.

2d. As to the best means of ventilating a sleeping-room, in which full one third of our entire existence is passed.

